# A topological analysis of difference topology experiments of condensin with topoisomerase II

**DOI:** 10.1242/bio.048603

**Published:** 2020-04-03

**Authors:** Soojeong Kim, Isabel K. Darcy

**Affiliations:** 1 Yonsei University, University College, Incheon 21983, South Korea; 2 Department of Mathematics, University of Iowa, Iowa City 52242, USA

**Keywords:** Difference topology experiment, DNA topology, Tangle analysis, Condensin

## Abstract

An experimental technique called difference topology combined with the mathematics of tangle analysis has been used to unveil the structure of DNA bound by the Mu transpososome. However, difference topology experiments can be difficult and time consuming. We discuss a modification that greatly simplifies this experimental technique. This simple experiment involves using a topoisomerase to trap DNA crossings bound by a protein complex and then running a gel to determine the crossing number of the knotted product(s). We develop the mathematics needed to analyze the results and apply these results to model the topology of DNA bound by 13S condensin and by the condensin MukB.

## Abstract (precis)

**Summary:** Tangles are used to model protein-DNA complexes. A three-dimensional ball represents protein while strings embedded in this ball represent protein-bound DNA. We used this simple model to analyze experimental results.

## INTRODUCTION

Proteins bind DNA in many genetic activities, such as replication, transcription, packaging, repair and rearrangement. Understanding the DNA conformation within protein-DNA complexes is useful for modeling and analyzing reactions (Crisona et al., 1999; Harshey and Jayaram, 2006; Kumar et al., 2017; Kimura et al., 1999; Pathania et al., 2002; Petrushenko et al., 2006). Laboratory techniques have been developed to study the shape of protein-DNA complexes including X-ray crystallography, cryogenic electron microscopy, atomic force microscopy (AFM) and nuclear magnetic resonance. Technology has significantly advanced, but it is still unsuccessful for large complexes in which proteins bind multiple DNA segments. To study protein-bound DNA, an experimental technique called difference topology combined with the mathematics of tangle analysis has been used (Kimura et al., 1999; Pathania et al., 2002; Petrushenko et al., 2006; Darcy et al., 2009; Kim and Darcy, 2015). Note that this technique focuses on determining the topology of DNA bound in a protein complex. Tangle analysis ignores the shape of the protein and cannot determine the exact geometry of the protein-bound DNA. But this simplified model has been used to determine reaction pathways and as a basis for more complex models (Darcy et al., 2008; Grainge et al., 2007; Harshey and Jayaram, 2006; Pathania et al., 2003; Shimokawa et al., 2013; Stolz et al., 2017; Vazquez et al., 2005). However, difference topology experiments can be difficult and time consuming.

In this paper we discuss a modification that greatly simplifies this experimental technique. We developed the mathematics needed to analyze the results. We applied these results to experiments performed on 13S condensins by Kimura et al. (1999) and the condensin MukB by Petrushenko et al. (2006).

**Fig. 1.**
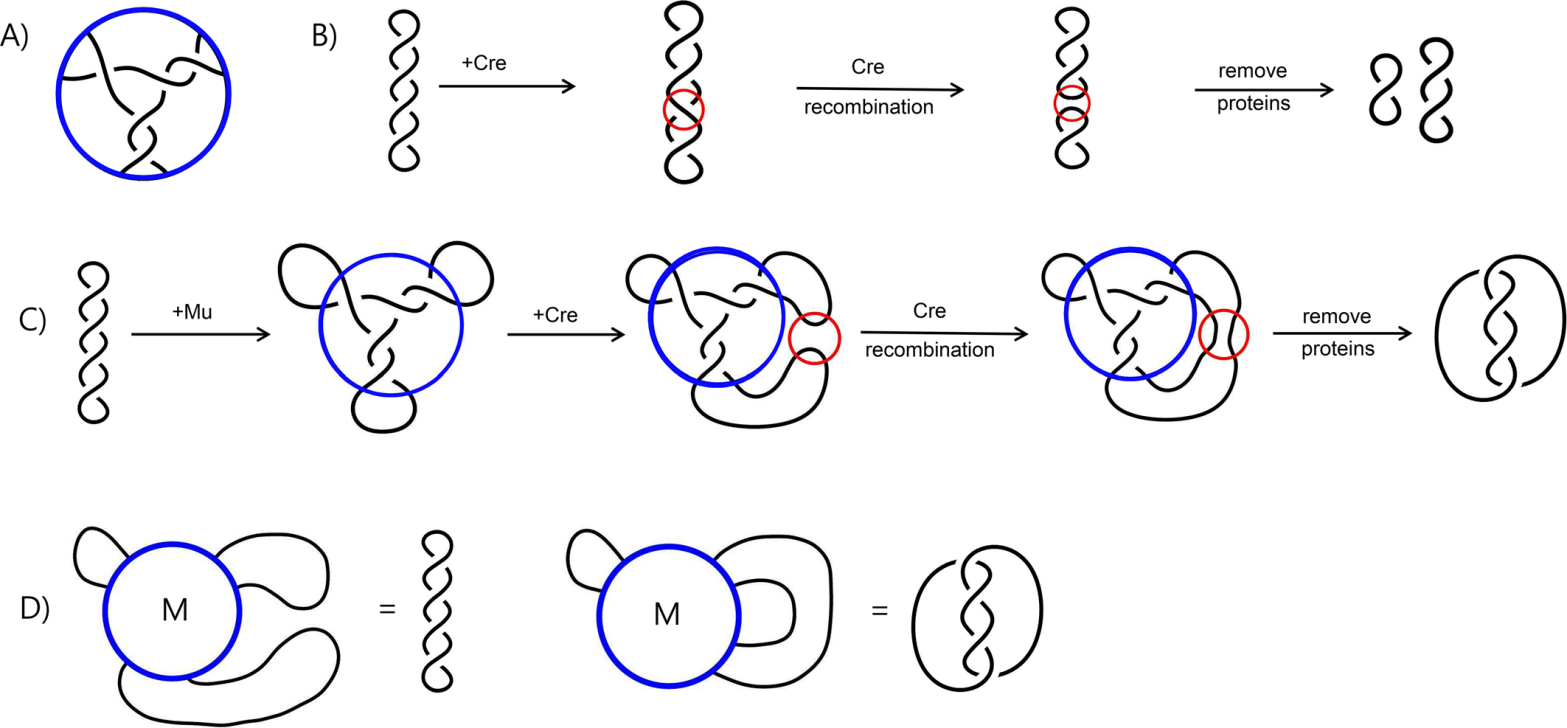
**Difference topology.** (A) The topology of DNA bound within the Mu transpososome. Note that Mu is represented by the blue circle and the three black lines represent the three DNA segments bound by Mu. (B) Cre acting on the DNA substrate. Cre is represented by the red circles. Note that the substrate is unknotted negatively supercoiled DNA. DNA is generally underwound and hence forms right-handed negative supercoils. The product of Cre recombination in this case is two smaller unlinked circles. (C) An example of a difference topology experiment from Pathania et al. (2002). Mu is represented by the blue circles while Cre is represented by the smaller red circles. Note that the product of Cre acting on DNA bound by Mu is a four-crossing catenane, while Cre acting on DNA without Mu produces two small unlinked DNA circles. This difference is the result of crossings bound by Mu that are trapped by Cre recombination. (D) Tangle equation modeling the reaction in C. The substrate equation is shown on the left and the product equation is shown on the right.

Tangles were used to model the biological results in Pathania et al. (2002). An *n*-string tangle is a three-dimensional ball with *n* strings properly embedded in it. When a protein complex binds DNA at *n* sites, the DNA-protein complex can be modeled by an *n*-string tangle (Ernst and Sumners, 1990). Fig. 1A shows the three-string tangle model for the Mu transpososome as determined in Pathania et al. (2002). The protein is modeled by the ball and the protein-bound DNA segments are modeled by the strings. Note that this is a two-dimensional model. However, two-dimensional tangle models can be used to create a three-dimensional tangle model (Vazquez et al., 2005).

Observe the difference in product topology between the reaction shown in Fig. 1B versus Fig. 1C. In Fig 1C, four of the five crossings bound by Mu are trapped via Cre recombination, resulting in a 4-crossing catenane; while in Fig 1B, Cre recombination results in two smaller unlinked circles. Difference topology experiments can be used to create a system of tangle equations with which one can solve the topology of the DNA bound by protein. The system of two tangle equations modeling the reaction in Fig. 1C is illustrated in Fig. 1D. The unknown variable *M* represents the tangle modeling the Mu transpososome before the tangle solution was determined. Before Cre recombination, the DNA is unknotted. After Cre recombination, the DNA product is a four-crossing catenane. Note that the tangle in Fig. 1A is one solution for the tangle variable *M* in this system of two tangle equations. However, this pair of equations is insufficient to determine a unique tangle solution modeling Mu. Thus Pathania et al. (2002) performed many additional experiments in order to create a system of nine tangle equations to solve for the unknown tangle *M*. Using only three of these equations, it was proved both mathematically (Darcy et al., 2009) and computationally (Darcy et al., 2006) that the model shown in Fig. 1A is the only biologically reasonable solution.

One issue with the difference topology technique using a recombinase such as Cre is the many experiments that need to be performed in order to create an accurate system of tangle equations. The sites for Cre can be placed in two different types of orientations, directly versus inversely repeated. The two different orientations are used to determine the topology of the outside loops [see Pathania et al. (2002) for more details]. Moreover, the location of binding sites for Cre must be determined. If the binding sites for Cre are placed too close to the binding sites for Mu, Cre will be unable to act. If the Cre binding sites are placed too far from the binding sites for Mu, recombination will trap extra DNA crossings, resulting in a variety of different types of DNA knots or catenanes. Thus, many experiments were performed in order to obtain unique products in each experiment used to create tangle equations. Next, we will discuss how topoisomerase can be used instead to greatly simplify this experimental technique.

## RESULTS

Instead of performing multiple experiments to generate a few tangle equations, one can perform a single experiment to generate multiple tangle equations using a type II topoisomerase instead of a recombinase. Type II topoisomerase will bind to one segment of DNA, break it, and allow a second segment to pass through this break before resealing the break. Thus, they can change DNA topology. Type II topoisomerases are not site specific; that is, they can bind anywhere along DNA. Using examples, we illustrate some modeling challenges and discuss the benefits of using a topoisomerase instead of a recombinase to trap crossings when performing difference topology experiments.

### Modeling two DNA segments bound by protein via difference topology using topoisomerase

**Fig. 2.**
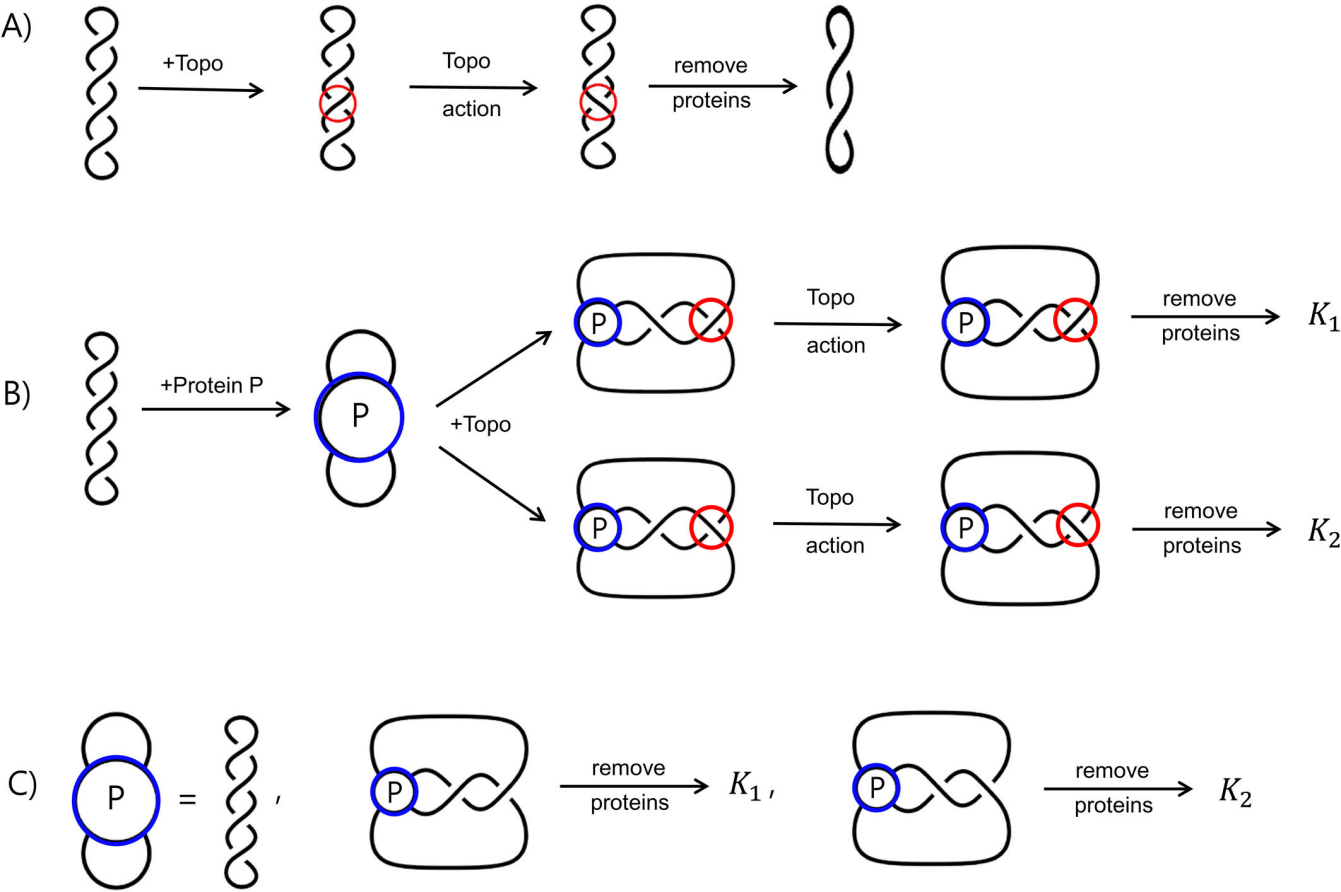
**Difference topology using a topoisomerase.** (A) Topoisomerase acting on unknotted supercoiled DNA. Topoisomerase is represented by the red circles. Reaction conditions are chosen so that topoisomerase does not create DNA knots. (B) An example of a difference topology experiment using a topoisomerase, in which *K*_1_ and *K*_2_ refer to the knotted products. The protein under study is modeled by the blue circles labeled *P* while topoisomerase is represented by the red circles. (C) Tangle equations modeling the reactions in B. The first equation corresponds to the unknotted substrate equation. The middle equation is the product equation, in which topoisomerase action is represented by a left-handed clasp. The last equation is the product equation, in which topoisomerase action is represented by a right-handed clasp.

Suppose we wish to study a protein that binds two segments of DNA as shown in Fig. 2, in which the protein under study is represented by the tangle *P*. Protein complexes that bind more than two DNA segments will be discussed in later sections. As before, we do both the control experiment in which the topoisomerase acts on naked DNA (Fig. 2A) as well as one in which protein *P* is added first so that it binds the DNA before topoisomerase is added (Fig. 2B). Note that the control experiment is very important to confirm that reaction conditions are such that topoisomerase action does not result in knotted DNA unless protein *P* has been added first. Topoisomerase under normal circumstances will unknot knotted DNA (Liu et al., 1980), but under some circumstances [such as in a difference topology experiment, but also under some reaction conditions (Wasserman and Cozzarelli, 1991)], topoisomerase will knot DNA. So the control experiment must be performed to ensure that the knots produced by topoisomerase in a difference topology experiment are due to the presence of protein *P* and not topoisomerase acting on naked DNA.

In Fig. 2B, the protein *P* is added first, binding DNA segments. Topoisomerase is then added to the reaction. The two loops come together forming two DNA crossings. Note that there are two different ways that the loops can come together as shown in this figure, resulting in two cases. Topoisomerase changes one of the two crossings. Without loss of generality, mathematically speaking, topoisomerase is shown acting on the crossing on the right in both cases. Three possible tangle equations modeling the reaction in Fig. 2B are shown in Fig. 2C. Note that we would still obtain these same tangle equations if topoisomerase acted on the crossing on the left in both cases. In the middle diagram of Fig. 2C, topoisomerase action is modeled by a left-handed clasp, while the diagram to the right shows a right-handed clasp. Whether or not topoisomerase action is modeled by a left-handed or right-handed clasp can be projection dependent as shown in Movie 1. For example, the two segments bound by topoisomerase could cross at a 90° angle. After topoisomerase action, if one views the three-dimensional model from the right, one would see a left-handed clasp, whereas if one viewed it from the left, one would see a right-handed clasp. Note that choosing a projection fixes the handedness of the clasp. Which projection we take also affects the two-dimensional tangle solutions for *P* (Vazquez et al., 2005). Thus, once we choose a particular projection to represent topoisomerase action (i.e. clasp handedness), we have also chosen a projection for the two-dimensional tangle *P*.

Whether we should use all three tangle equations shown in Fig. 2C depends on the three-dimensional conformation of the DNA loops emanating from the protein complex. Consider first the unknotted substrate equation (the first equation in Fig. 2C). In this equation showing the unknotted substrate, none of the three loops interact. This is the same assumption that was made by Pathania et al. (2002) when using Cre recombinase to determine the DNA conformation within the Mu transpososome. This is a reasonable assumption as illustrated in Movie 2. Depending on how the three-dimensional protein-DNA complex is projected, we may or may not see two of the loops cross. In most cases, there should be a projection in which the loops do not cross as assumed in Fig. 2C. However, if it is later determined that no such projection exists, the solution found using the outside loop configuration shown in Fig. 2C can be easily modified to satisfy a different configuration of outside loops (Darcy et al., 2006, 2009). Thus, we will always assume that when protein *P* binds DNA, the loops emanating outside the protein-DNA complex do not cross in the substrate equation (Figs 1D and 2C, and Eqn 1 in Table 1).

**Table 1.**
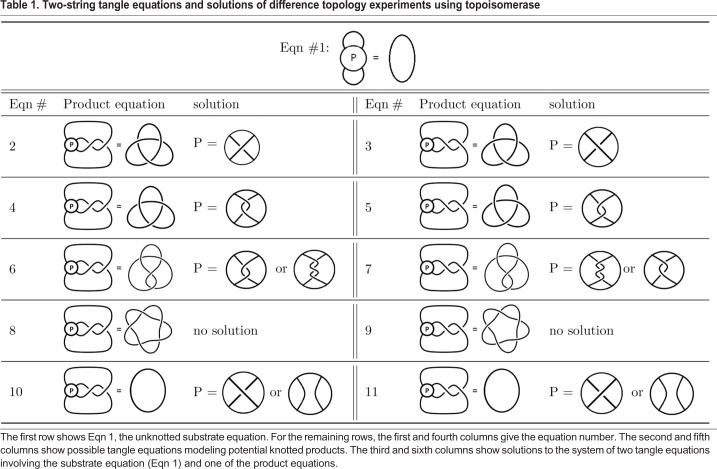
**Two-string tangle equations and solutions of difference topology experiments using topoisomerase**

The main question is whether we should use both of the product equations. In one case, topoisomerase creates a right-handed clasp. In the other case, topoisomerase creates a left-handed clasp. How the loops emanate from the protein-DNA complex could make one configuration more likely than the other one. Thus, one must allow for some ambiguity in terms of which tangle equations best model the difference topology experiment. However, we can still gain useful information despite this ambiguity.

Table 1 shows solutions to systems of two tangle equations, where the first equation is always the unknotted substrate equation shown at the top of this table and the second equation is one of the product equations. One can also use the TopoICE-X (Darcy et al., 2008) and TopoICE-R (Darcy and Scharein, 2006) software within KnotPlot (R. G. Scharein, Interactive topological drawing, PhD thesis, The University of British Columbia, 1998) to solve most biologically relevant two-string tangle equations. We will illustrate how Table 1 can be used with the examples below. In these examples, we will sometimes assume we only know the crossing number of the knotted product as this can be determined via gel electrophoresis. The crossing number of a knot is the smallest number of crossings needed to draw that knot. The unknot is the only knot that can be drawn with fewer than three crossings. There are two three-crossing knots and one four-crossing knot (shown in Table 1). For a table that includes higher crossing knots, please see Rolfsen (1976) and https://knotplot.com/zoo/.

Example 1. Suppose that the products observed are three-crossing and four-crossing knots. Thus assuming protein *P* binds a unique DNA conformation, we have a system of three tangle equations (as in Fig. 2C): one for the unknotted substrate and one each for the two products. From Table 1, these equations must be Eqns 1, 5 and 6 or Eqns 1, 4 and 7. If we look at any other combination of equations, we do not have a common solution. For example, Eqns 1 and 2 mean that protein *P* must bind one crossing. However, if *P* binds only one crossing, topoisomerase action cannot produce a four-crossing knot as per Eqns 6 and 7. As per these equations, protein *P* must bind to two or three crossings in order to produce a four-crossing knot when starting with an unknotted substrate. Thus, Eqn 2 cannot be one of the equations modeling this reaction if protein *P* binds a unique DNA conformation. However, the system of three tangle Eqns 1, 5 and 6 has a solution whereby protein *P* binds two negative supercoils. Note that this two-crossing solution satisfies the system of two tangle Eqns 1 and 5 as well as the pair of tangle Eqns 1 and 6 and thus satisfies all three Eqns 1, 5 and 6. If Eqns 1, 4 and 7 model these reactions, then protein *P* binds to two positive supercoils.

One should also consider the possibility that one of these products is the result of multiple rounds of topoisomerase action. For example, topoisomerase could act twice on the unknotted substrate to produce the four-crossing knot. However it is not possible to convert a three-crossing knot to a four-crossing knot (or vice versa) via a single topoisomerase action (Darcy and Sumners, 1997; Torisu, 1998; Darcy et al., 2008). Since no other knot types were detected, it is unlikely that topoisomerase acted more than once on the unknotted substrate to produce the four-crossing knot (and similarly for the three-crossing knot).

Example 2. Suppose that the only products are three-crossing knots. The tangle equations modeling this reaction would be the substrate equation (Eqn 1 in Table 1) along with only one of the Eqns 2–5. Any system of three equations involving Eqn 1 and two equations from Eqns 2–5 has no solution as these equations do not have a solution in common. Thus, only one of Eqns 2-5 can hold. Hence, we can only determine that protein *P* binds to one or two crossings. If we wish to determine the handedness of these crossings, one would need to identify whether the three-crossing knots are right-handed or left-handed via AFM or electron microscopy (EM). But since only one of these equations can hold, that means that topoisomerase action in this case must result in either the left-handed or right-handed clasp, but not both. Hence, we can infer that the loops must emanate from protein *P* in such a manner that only one-handedness is possible.

Example 3. Suppose that one of the products is the five-crossing knot shown in Eqns 8 and 9. Topoisomerase must act twice on unknotted DNA to produce this five-crossing knot. Thus, there is no solution to the system of two-tangle Eqns 1 and 8 as well as Eqns 1 and 9. We can often distinguish between products that require two or more rounds of topoisomerase action from those that can be produced via a single-crossing change via topoisomerase action (Darcy and Sumners, 1997; Torisu, 1998; Darcy et al., 2008). However, knotted products that require multiple rounds of topoisomerase action can still be used for modeling. We will discuss this example further when we analyze experiments involving condensins.

Example 4. Suppose that the only knot types detected are unknots. Then it is possible that the shape of DNA bound by protein is simple as per Eqns 1 and 10/11, in which the protein complex binds at most one crossing. However, it is also possible that the protein complex under study did not stably bind DNA and thus the reaction shown in Fig. 2B may not have occurred. Thus if no knots are detected, one cannot make any conclusions regarding the DNA conformation bound by protein.

### Modeling three DNA segments bound by protein via difference topology using topoisomerase

**Fig. 3.**
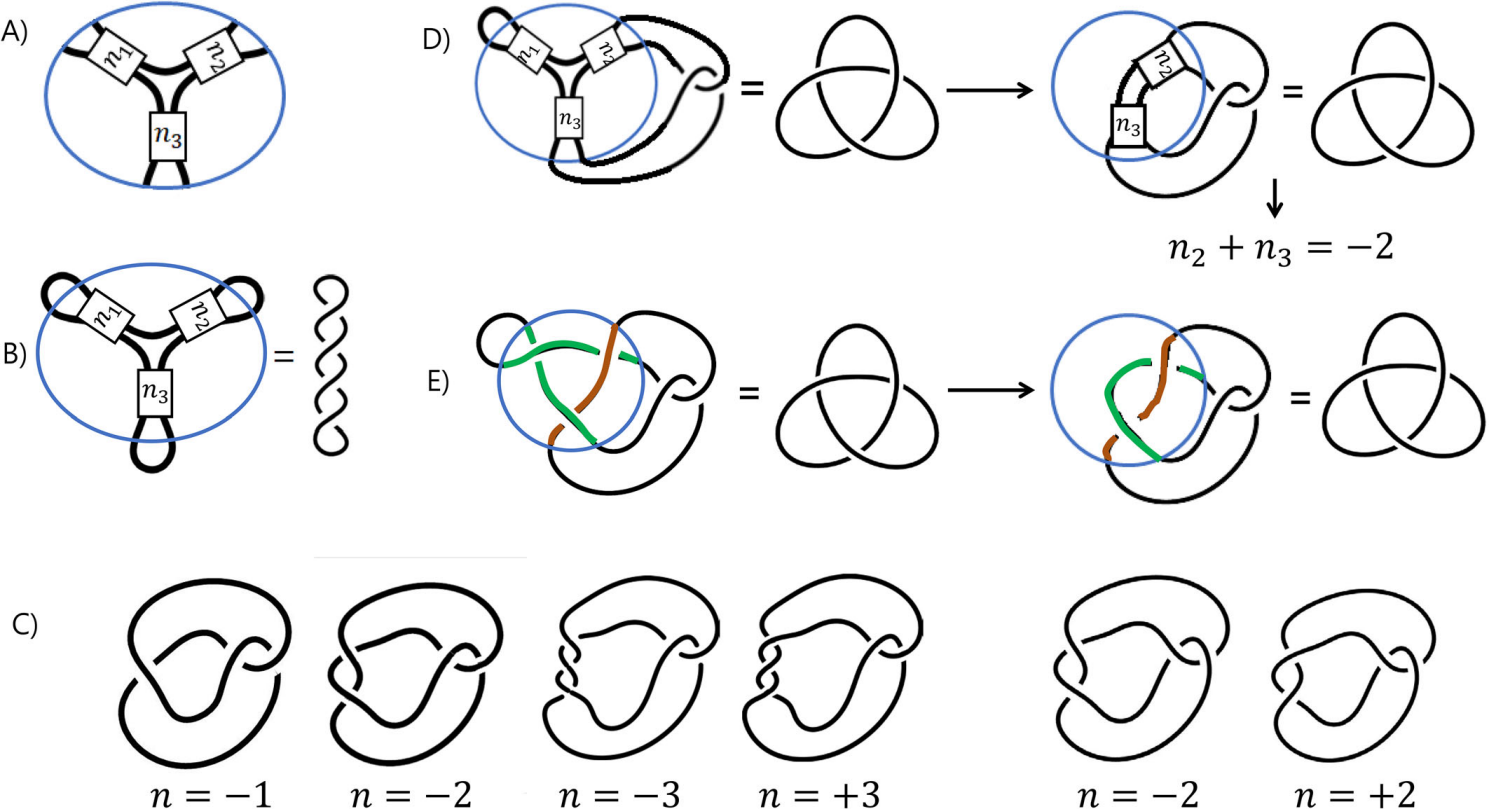
**Modeling three DNA segments bound by protein via difference topology using topoisomerase.** (A) A protein complex binding three DNA segments that form a three-branched supercoiled DNA, in which *n*_*i*_ represents the number of supercoils in the *i*th branch. The integer *n*_*i*_ is negative if intertwining is right-handed (and thus represents negative supercoils). If the intertwining is left-handed, then *n*_*i*_ is positive (and thus represents positive supercoils). (B) Unknotted substrate equation assuming that the protein complex binds a three-branched supercoiled structure. (C) A twist knot is formed by trapping crossings via a clasp. If the clasp is left-handed and *n*=−1, then the knot diagram can be simplified to contain no crossing and thus we have the unknot. If *n*=−2, then the diagram can be simplified to the right-handed three-crossing knot. If *n*=−3, then the diagram simplifies to the four-crossing knot, while if *n*=3, we obtain a five-crossing twist knot. If the clasp is right-handed, then if *n*=−2, we obtain the four-crossing knot, while if *n*=2, then the diagram simplifies to the left-handed three-crossing knot. (D) A three-string tangle equation can be transformed into a two-string tangle equation by pushing one of the outside loops into the tangle ball. In the case shown, we can then remove the supercoils in the branch containing *n*_1_ supercoils, leaving *n*_2_+*n*_3_ supercoils. (E) An example of a solution to the three-string tangle equation in D, where *n*_1_=*n*_2_=*n*_3_=−1. Note that since *n*_2_+*n*_3_=−2, the brown DNA segment crosses the green DNA segments two times.

The DNA within the Mu transpososome is modeled by a three-branched supercoiled structure as shown in Fig. 1A. A more general three-branched structure is shown in Fig. 3A, in which *n*_*i*_ represents the number of supercoils in the *i*th branch. Note that this solution satisfies the unknotted substrate equation for any choice of integers *n*_1_, *n*_2_, *n*_3_ as shown in Fig. 3B. If topoisomerase acts on a pair of loops emanating from a three-branched structure, then the product will likely be a twist knot, a knot in which supercoils are trapped by a clasp, as shown in Fig. 3C.

As per proof in mathematical methods, if topoisomerase acts on each pair of loops emanating from a protein complex binding three DNA segments producing twist knots with less than 100,000 crossings, then the only biologically plausible model for the DNA bound within the protein complex is a three-branched supercoiled structure as shown in Fig. 3A. If knot types other than twist knots are produced via a single round of topoisomerase action, then the configuration will be more complicated. Potential models in this case can be determined computationally (Darcy et al., 2006). Thus, we will focus on twist knot products.

Example 5. Consider the tangle equation in Fig. 3D, in which the product is the right-handed three-crossing knot. Since topoisomerase acts on the loops on the right, the knotted product has *n*_2_+*n*_3_ supercoil crossings between the clasp, while the *n*_1_ crossings in the loop on the left can be ‘mathematically removed’ – i.e., removing these *n*_1_ crossings does not change the knot type of the product. After the protein complex is removed, the knot diagram can be simplified to a diagram with only three crossings. One solution to the tangle equation is shown in Fig. 3E. In this figure, we chose *n*_1_=*n*_2_=*n*_3_=−1. We could have chosen any value for *n*_1_ and any pair of values for *n*_2_ and *n*_3_ satisfying *n*_2_+*n*_3_=−2. We know that *n*_2_+*n*_3_=−2 from Eqns 1 and 3 in Table 1. By removing the branch on the right, we have converted the three-string tangle equation (Fig. 3D, left) into a two-string tangle equation (Fig. 3D, right). Thus we can use Table 1 Eqn 3 to determine that *n*_2_+*n*_3_ must represent two negative supercoils and thus *n*_2_+*n*_3_=−2. This also tells us how two of the three DNA segments interact. In Fig. 3E, the brown DNA segment must cross the green DNA segments twice.

If we have three tangle equations, one for each pair of loops, that will give us three independent linear equations involving *n*_1_, *n*_2_, *n*_3_, for which there will be a unique solution. However, we do not actually know which system of tangle equations best models difference topology experiments involving a topoisomerase. In particular, perhaps topoisomerase action results in the left-handed clasp (Fig. 2C, middle) instead of the right-handed clasp (Fig. 2C, right). In that case, Eqn 2 in Table 1 implies that *n*_2_+*n*_3_=−1. Suppose that the right-handed three-crossing knot shown in the tangle equation Fig. 3D is the only product of a difference topology experiment involving topoisomerase. Suppose further that topoisomerase acted on every pair of loops, producing only right-handed three-crossing knots. If we do not assume which clasp, we obtain the following system of equations: (*n*_1_+*n*_2_=−2 or *n*_1_+*n*_2_=−1) and (*n*_1_+*n*_3_=−2 or *n*_1_+*n*_3_=−1) and (*n*_2_+*n*_3_=−2 or *n*_2_+*n*_3_=−1). We are only interested in integer solutions since in a projection, we cannot have a fractional crossing. The integer solutions to this system of equations are (*n*_1_, *n*_2_, *n*_3_)=(−1,−1,−1), (−1,−1, 0), (−1, 0,−1), (0,−1,−1). All these solutions could correspond to different projections of the same three-dimensional model.

### Multiple DNA segments bound by protein via difference topology using topoisomerase

**Fig. 4.**
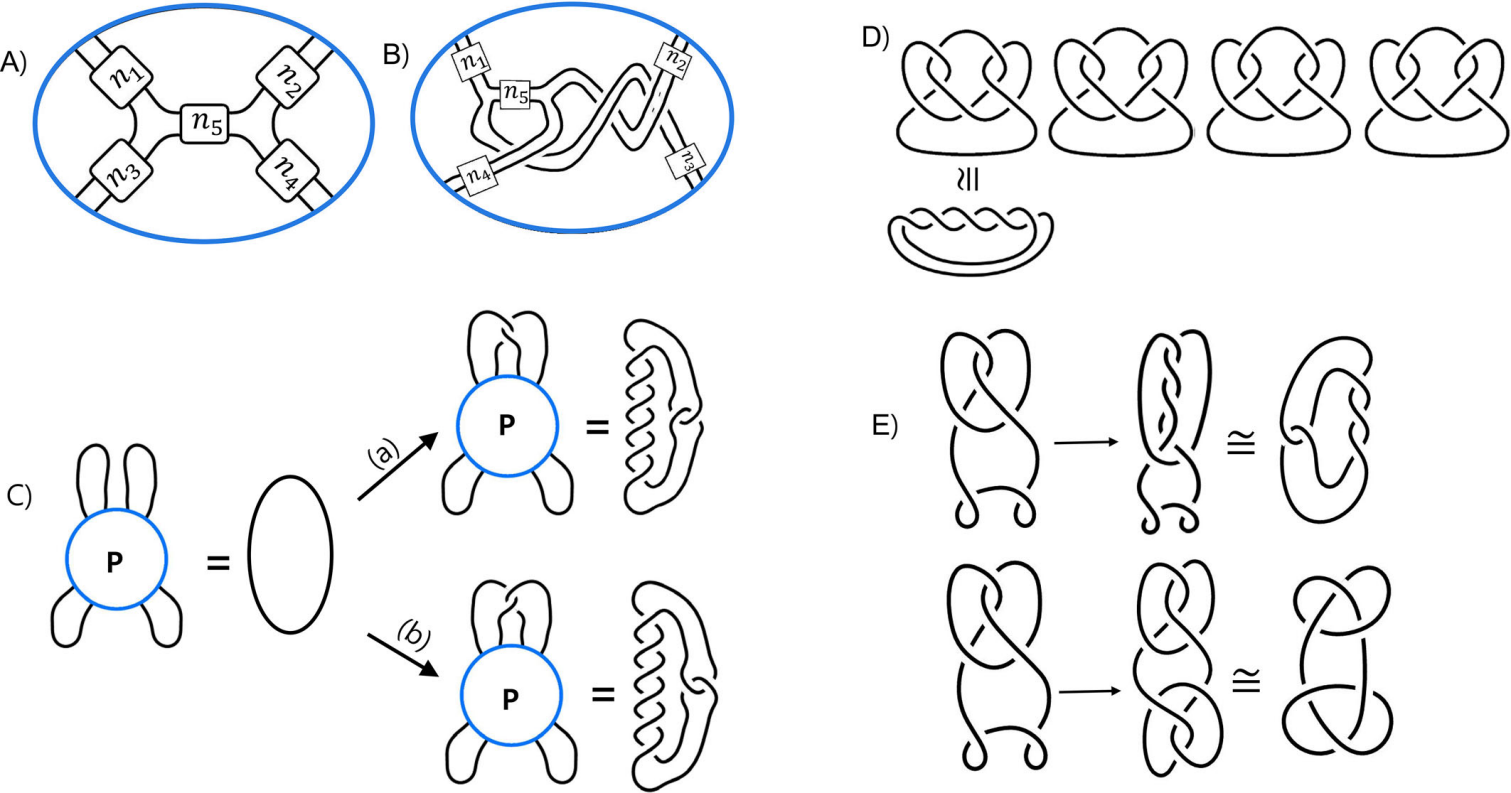
**Modeling four DNA segments bound by protein via difference topology using topoisomerase.** (A) A possible branched supercoiled DNA structure in which the protein complex has bound four DNA segments. This conformation is referred to as 0-standard. The number of half twists in each branch is denoted by *n*_*i*_, where the half twists are left handed if *n*_*i*_>0 (representing positive supercoils) and right-handed if *n*_*i*_<0 (representing negative supercoils). (B) A more complicated branched supercoiled DNA structure. This type of conformation with be referred to as R-standard. (C) Four-string tangle equations modeling difference topology experiments with type II topoisomerase. The tangle *P* represents the four-string tangle model of protein-bound DNA. Topoisomerase action will result in either (a) a left-handed clasp or (b) a right-handed clasp. (D) If topoisomerase acts on three consecutive loops, other knot types can be obtained. From left to right: if two left-handed clasps are created, then the result is a five-crossing torus knot. If one left-handed clasp and one right-handed clasp are formed, then the product is a six-crossing knot (middle two diagrams). If both clasps are right-handed, then the product is a seven-crossing knot. (E) If topoisomerase acts twice on the same pair of loops, the result is a five-crossing twist knot (top row). If topoisomerase acts on two different pairs of loops, then the result is the six-crossing granny knot (bottom row).

If a protein complex *P* binds four DNA segments, then a branched supercoiled structure might look like that in Fig. 4A. This configuration will be referred to as 0-standard. However, it is possible that the structure is more complicated as shown in Fig. 4B. This configuration will be referred to as R-standard. An R-standard tangle can be created from a 0-standard tangle by intertwining the branches containing *n*_1_, *n*_3_, *n*_4_ supercoils. For example, the tangle in Fig. 4B was created from the 0-standard tangle by first twining the branches containing *n*_3_ and *n*_4_ supercoils once, followed by twining the branches containing *n*_2_ and *n*_4_ supercoils twice. As proved in the ‘Mathematical methods’ section, if topoisomerase acts on each pair of loops emanating from a protein complex binding four DNA segments producing twist knots with less than 100,000 crossings, then the DNA bound within the protein complex must be R-standard. Since 0-standard is more biologically relevant, we will focus on 0-standard solutions.

We will use four-string tangle analysis to analyze experiments involving condensins. Note that condensins bind multiple DNA segments. However, one can use four-string tangle analysis to determine how any quadruple of strands interact. Moreover, often one can partition an *n*-string tangle model into smaller four-string tangle models.

### Condensins and tangle equations involving multiple DNA segments

Condensins are large protein complexes that play a major role in chromosome assembly and segregation during meiosis and mitosis. Condensins interact with multiple sites of DNA to construct the structure of the chromosome (Petrushenko et al., 2006; Uhlmann, 2016). The 13S condensin is a eukaryotic condensin from *Xenopus*. To understand how 13S condensin interacts with DNA, Kimura et al. used the difference topology experimental technique with type II topoisomerase (Kimura et al., 1999). Similar experiments were performed with MukB, the first discovered bacterial condensin in *Escherichia coli*, by Petrushenko et al. (2006). But they did not formally describe tangle equations modeling these reactions as the modeling is more complex for difference topology experiments and the mathematics for solving such equations did not exist until now.

Based on single-molecule experiments (Ganji et al., 2018) and computational modeling (Orlandini et al., 2019; Racko et al., 2018), it is believed that condensins work with topoisomerase to unknot DNA via loop extrusion. However, difference topology experiments are usually performed under equilibrium conditions in which the protein under study is believed to be stably bound to DNA, while topoisomerase is used to knot the DNA by trapping crossing bound by the protein understudy. Moreover, in both the 13S condensin and MukB difference topology experiments, a nicked unknotted DNA substrate was used. This means that the DNA was not supercoiled. In the presence of type II topoisomerase and 13S condensin, the main product topology of DNA was the right-handed three-crossing knot. In addition, they found a few four-, five- and six-crossing knots. But, as measured by gel electrophoresis, the three-crossing knots were far more abundant than the higher crossing knots. For MukB, the right-handed three-crossing knot was also the main knot type produced in the difference topology experiment. But in this case, they also found a fair amount of four-crossing knots and some five-crossing knots.

To determine the handedness and knot type of the higher-crossing products, some of the knots were identified via EM. Note that EM does not quantify the amount of each knot type, but can be used to determine which knot type is more common among knotted products having the same crossing number if a sufficient number of knots are identified. For 13S condensin, of the three-crossing knots identified, 130 were right-handed while only eight were left-handed (Kimura et al., 1999). For MukB, 37 right-handed three-crossing knots were identified while only one left-handed three-crossing knot was observed (Petrushenko et al., 2006). Thus, since most knots were determined to be three-crossing knots via gel electrophoresis, and the vast majority of these knots were identified as right-handed via EM, we know that the main product of topoisomerase action on DNA bound by condensin are right-handed trefoil knots in both of these experiments.

### An *n*-string tangle analysis of condensin-DNA complexes

Condensins bind DNA at multiple sites, so condensin-DNA complexes can be modeled by an *n*-string tangle, where *n* is a large number. We will focus on *n*=4 since an *n*-string tangle model can sometimes be partitioned into four-string tangle models. There are various hypothetical models of condensin-DNA complexes (Eeftens et al., 2017; Ganji et al., 2018; Hirano, 2012; Hayama et al., 2013; Kumar et al., 2017; Kimura et al., 1999; Losada and Hirano, 2005; Petrushenko et al., 2006, 2010; Rybenkov et al., 2014). Many of these models show a four-string tangle model (for example Fig. 7 in Petrushenko et al., 2006), as these models can be easily be extended to *n*-string tangle models.

We will look at two cases. For the first case, we will assume that the product of one round of topoisomerase action produces only right-handed trefoil knots. This is likely the case in the 13S condensin experiments, as this was by far the major product based on gel electrophoresis and EM. For the second case, we will also assume that the right-handed trefoil knot is also the major product, but that products also included a fair number of four-crossing knots as per the MukB experiments. Other products will be explained below in the minor products subsection. We focus on 0-standard solutions as other solutions are more complicated and hence unlikely to be biologically relevant.

### 13C condensin

Suppose that 13S condensin binds DNA in a 0-standard configuration (Fig. 4A), and topoisomerase action on each pair of loops results in the right-handed trefoil knot. In this case, at least three of four branches (corresponding to *n*_1_, *n*_2_, *n*_3_, *n*_4_) contain one positive supercoil with the remaining branch containing one positive or no supercoils, while the middle branch (corresponding to *n*_5_) will have one negative or no supercoil. These solutions can all be seen in the three-dimensional model shown in Movie 3.

Note that this solution is consistent with the models proposed in Petrushenko et al. (2006), but we have additional information. If the solution is non-planar, then the twisting between branches should be right-handed since *n*_5_=0,−1.

### MukB

Suppose that MukB binds DNA in a 0-standard configuration (Fig. 4A), and topoisomerase action on each pair of loops results mostly in the right-handed trefoil knot as well as a fair amount of four-crossing knots. If we restrict to the case where we assume at most one-third of the product consists of four-crossing knots and the remaining products are right-handed trefoil knots, then there are 84 solutions. Several two-dimensional solutions can be visualized via a single three-dimensional solution, but we would still have a number of possible three-dimensional solutions if we just considered mathematics. However, from a biological perspective, simpler solutions are likely to be more relevant. The simplest solution would be a modification of the solution we found for 13S condensin.

Consider the conformation shown in Movie 3. For topoisomerase action to result in the three-crossing knot, the crossing change must result in the left-handed clasp (top pathway in Fig. 4C). In Petrushenko et al. (2006), it was hypothesized that a major conformation change was responsible for the four-crossing knotted product, but a conformation change is not needed. If topoisomerase action results in a right-handed clasp (bottom pathway in Fig. 4C), then a four-crossing knot will be produced. The topoisomerase used in Kimura et al. (1999) and Petrushenko et al. (2006) does not have a chirality bias. Thus, the fact that three-crossing knots are more abundant than four-crossing knots means that the loops must extrude from the protein complex in a manner such that a left-handed clasp is more likely to form than a right-handed clasp. This is particularly true for 13S condensing, in which the three-crossing products were 20-fold more common than the four-crossing products as detected via gel electrophoresis. Since four-crossing products are more common for MukB (although still less than trefoils), this means that while both three-dimensional models may project to the same configuration, there is a difference between their three-dimensional configurations. Computational looping studies could be used to investigate three-dimensional models more thoroughly (Vetcher et al., 2006).

### Minor products

Note that the model in Movie 3 can also be used to explain the minor products in the MukB and 13S condensin experiments. In both these experiments, a small number of five- and six-crossing knots were observed via gel electrophoresis (Kimura et al., 1999; Petrushenko et al., 2006). A few of five- and six-crossing knots were identified via EM in Kimura et al. (1999). Nine of the identified five-crossing knots were right-handed five-crossing torus knots (Fig. 4D, leftmost diagram). Four five-crossing twist knots were also identified, one with the handedness shown in Fig. 4E (top row) and three with the opposite handedness. All five of the six-crossing knots that were identified were right-handed granny knots (Fig. 4E, bottom row). Since only a few five- and six-crossing knots were identified, we do not have a statistically significant sample size to infer which of these minor products were more common. It is mathematically impossible for topoisomerase to create five-crossing torus knots or six-crossing granny knots by acting only once on an unknotted substrate (Darcy and Sumners, 1997; Torisu, 1998). The five-crossing torus knot will result if topoisomerase action occurs on two pairs of three adjacent DNA loops as in Fig. 4D (leftmost diagram). If topoisomerase acts on two pairs of loops involving four DNA loops as in Fig. 4E (bottom row), the result is the six-crossing granny knot. While the five-crossing twist knot could be the result of a single round of topoisomerase action, it could also be the result of a second round of type II topoisomerase action on the same pair of loops used in the first round (see Fig. 4E, top row).

Note that for these knots, topoisomerase action results in left-handed clasps. If topoisomerase action also created right-handed clasps, then six-crossing knots and seven-crossing knots can be obtained as shown in Fig. 4D. However, those knots are not observed in the difference topology experiments of condensin-DNA complexes (Kimura et al., 1999; Petrushenko et al., 2006), supporting the preference for the formation of the left-handed clasp [Fig. 4C(a)] over the right-handed clasp [Fig. 4C(b)] via topoisomerase action.

Some knots could also be the result of topoisomerase action on naked DNA. While knots were not detected in the control reaction in which topoisomerase acted on naked DNA, gel electrophoresis may not detect all knots. There could be some bands containing knots that are not visible via Southern blotting. Thus, the small number of left-handed trefoils and five-crossing twist knots could result from topoisomerase action on naked DNA.

## DISCUSSION

While tangle equations related to difference topology experiments with Cre have been well studied (Darcy et al., 2006, 2009; Harshey and Jayaram, 2006; Pathania et al., 2002, 2003; Yin et al., 2005, 2007), difference topology experiments using type II topoisomerase have not yet been actively studied. We analyzed the difference topology experiments of condensin-DNA complexes with type II topoisomerase (Kimura et al., 1999; Petrushenko et al., 2006) using the tangle model and conclude that the conformation shown in Movie 3 is the most biologically relevant tangle model for condensin-DNA complexes. This conclusion also supports the working model suggested by Petrushenko et al. (2006).

Note that in both the model in Petrushenko et al. (2006) and our model based on the data in Petrushenko et al. (2006), condensin binds to positive supercoils. If condensin bound only to negative supercoils, then topoisomerase would not have produced right-handed three-crossing knots. When a protein complex binds DNA, the DNA may partially unwind where the protein complex binds the DNA, resulting in local undertwisting of this protein-bound DNA. DNA in the cell is generally negatively supercoiled. This negative supercoiling allows for proteins to more easily untwist the two strands of double-stranded DNA. When this occurs, the negative supercoiling is converted to underwound DNA. Thus, the amount of negative supercoiling decreases. This can occur via a decrease in the number of supercoils or in the creation of a positive supercoil. When condensin binds a DNA loop, the local untwisting of DNA can result in a positive supercoil in that loop. Condensin bound to that positive supercoil would trap that supercoil and thus topoisomerase action results in a right-handed three-crossing knot.

If the change in DNA twist caused by the binding of condensin is offset by the creation of a positive supercoil bound by condensin, then there would be no change in the overall supercoiling of the DNA. But the supercoiling bound by protein need not match the change in twist caused by protein binding. For both MukB (Petrushenko et al., 2006) and 13S condensin (Kimura et al., 1999), a net change in supercoiling was observed. In the case of MukB, the net change was negative, while for 13S condensin, the net change was positive. Recall that the middle branch in the 0-standard configuration will contain either zero or one negative supercoil (represented by *n*_5_ in Fig. 4A). If the middle branch contains a negative supercoil, this could explain the net negative change in supercoiling for MukB.

The tangle equations give us possible two-dimensional models for protein-bound DNA. As per the movies, this is one reason that we often obtain more than one solution to systems of tangle equations modeling difference topology experiments involving a topoisomerase: the actual three-dimensional model can project to more than one two-dimensional tangle solution. In particular the three-dimensional conformation of DNA is unlikely to have exactly zero or exactly one negative supercoil in the middle branch represented by *n*_5_. Thus, in three dimensions, *n*_5_ would be better represented by a fraction between 0 and −1. We hypothesize that for MukB, the three-dimensional conformation of DNA bound in the MukB-DNA complex contains a middle branch with a fractional negative supercoil closer to one, while for 13S condensin this fractional negative supercoil would be closer to zero. This would explain both the difference in knotted products as well as the net change in supercoiling (negative for MukB and positive for 13S condensin).

The main advantage of difference topology experiments using a topoisomerase is that one can determine the feasibility of this experimental technique applied to a particular protein complex without a significant investment of time. If one uses a site-specific recombinase, one must create substrates with recombinase binding sites correctly placed on every pair of loops. If one uses a topoisomerase, one can simply add topoisomerase to a test tube containing the protein complex under study using reaction conditions where this protein complex stably binds DNA. The control reaction also must be performed where topoisomerase acts on naked DNA under identical reaction conditions except for the omission of the protein complex under study. If there is a difference in the knot types of the products as determined via gel electrophoresis, then difference topology can give insight into the conformation of DNA bound by protein. Moreover, depending on difference topology experimental results using a topoisomerase, one can determine whether one is likely to obtain sufficiently better information using a recombinase instead of a topoisomerase. One can also use the difference topology results using a topoisomerase to predict the results if one were to use a recombinase instead (Price and Darcy, 2019).

Of course, experimental procedures are rarely simple. One may need to play around with reaction conditions. In particular, one might need to use a singly nicked DNA substrate as was used in the condensin experiments. If supercoiled DNA is used, then topoisomerase may trap supercoils not bound by protein. One can use the simplest products to determine the minimal complexity of DNA bound by protein, but results involving multiple different types of DNA knots would be much harder to analyze. Using a single nicked DNA may reduce the number of knot types resulting from topoisomerase action. However, the use of a nicked DNA substrate can affect the DNA conformation bound by DNA. Recall that difference topology was used to determine that Mu transposase binds to five DNA crossings as per the tangle model in Fig. 1A. In this case, a supercoiled DNA substrate was used. When difference topology with Cre recombinase was applied to Mu transposase binding to nicked DNA, Mu transposase only bound to four DNA crossings instead of five (Yin et al., 2005).

While analyzing difference topology experiments involving a topoisomerase is not as straight forward as analyzing difference topology experiments involving a site-specific recombinase, the results can still be very useful for modeling the three-dimensional conformation of DNA bound by protein as we have illustrated with our analysis of 13S condensin and MukB. When tangle equations have been used to model a difference topology experiment using a site-specific recombinase, only one biologically relevant two-dimensional tangle solution has been found (Harshey and Jayaram, 2006; Pathania et al., 2002, 2003; Yin et al., 2005, 2007). The ambiguity in the tangle model for difference topology experiments involving a topoisomerase means we do not expect to find only one biologically relevant two-dimensional tangle solution. But this ambiguity led us to consider three-dimensional models consistent with the two-dimensional tangle solutions. So although difference topology experiments involving a recombinase are less ambiguous and easier to analyze, we obtain better three-dimensional analysis when a topoisomerase is used instead.

## MATERIALS AND METHODS

### Mathematical methods

Most two-string tangle equations modeling topoisomerase action can be solved using TopoICE-X (Darcy et al., 2008) in KnotPlot (R. G. Scharein Interactive topological drawing, PhD thesis, The University of British Columbia, 1998), including all equations in which the knots involved have fewer than eight crossings.

We use the following theorem to solve three- and four-string tangle equations:

Theorem 1. Suppose that protein *P* binds DNA and topoisomerase acts on all pairs of loops producing only twist knots with fewer than 100,000 crossings. Then the only biologically relevant tangle model representing *P* is a three-branched structure (Fig. 1A) if protein *P* binds to three DNA segments or R-standard if protein *P* binds to four DNA segments.

Proof. Consider the tangle equation modeling topoisomerase acting on one pair of loops emanating from the protein *P*-DNA complex. We can convert this tangle equation into a two-string tangle equation by pushing all loops except the pair of loops upon which topoisomerase acted into the tangle ball (see, for example, Fig. 3D). We can solve the two-string tangle equation using results in Darcy and Sumners (1997) and Torisu (1998), in which the product of topoisomerase acting on unknotted DNA is a twist knot. We solved all such equations in which the twist knot had fewer than 100,000 crossings by writing a simple C program (code available upon request). This allows us to use the results in Darcy et al. (2009) and Kim and Darcy (2015) to prove that the only biologically relevant tangle model representing *P* is a three-branched structure if *P* binds three DNA segments or R-standard if *P* binds to four DNA segments.

## Supplementary Material

Supplementary information
