# Supplementary material for: A topological analysis of difference topology experiments of condensin with topoisomerase II
Source: Biol Open. 2020 Apr 3;9(4):bio048603. doi: 10.1242/bio.048603 (PMC7132813; doi:10.1242/bio.048603)
Supplement: Supplementary information [file biolopen-9-048603-s1.pdf]

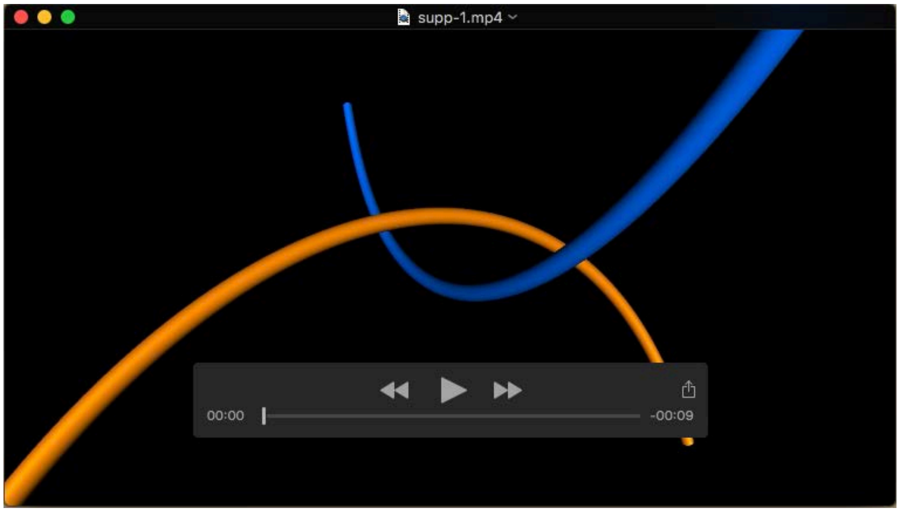

Movie 1: Left-handed vs right-handed clasp. The clasp first appears as a left-handed, but viewing the clasp from the other side, the clasp appears as a right-handed clasp. Thus whether or not a clasp is right-handed or left-handed is projection dependent. In this movie the two segments cross at a 90 degree angle. But if the two segments cross at a different angle, than one handedness will appear in a larger percentage of projections than the other handedness.

Biology Open • Supplementary information

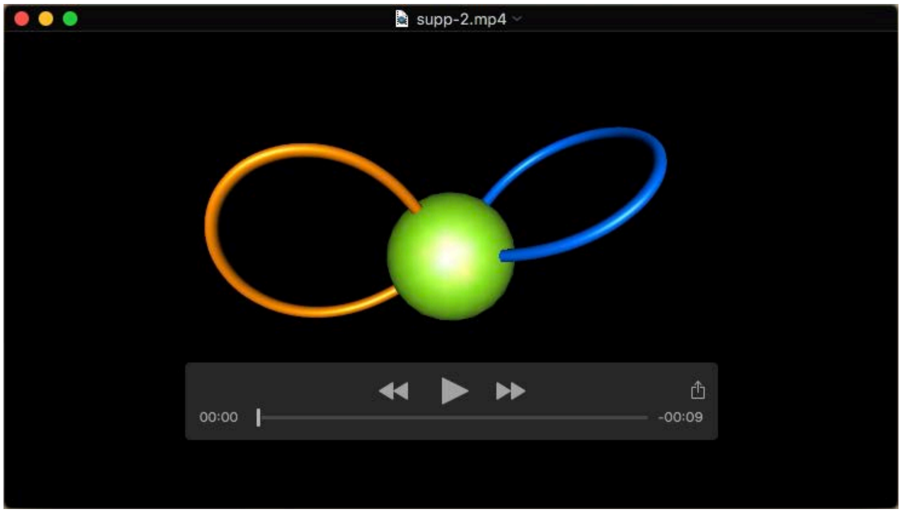

Movie 2: Exterior loops. Depending on how one projects a 3-dimensional protein-DNA complex, the two outside loops may or may not cross.

Biology Open • Supplementary information

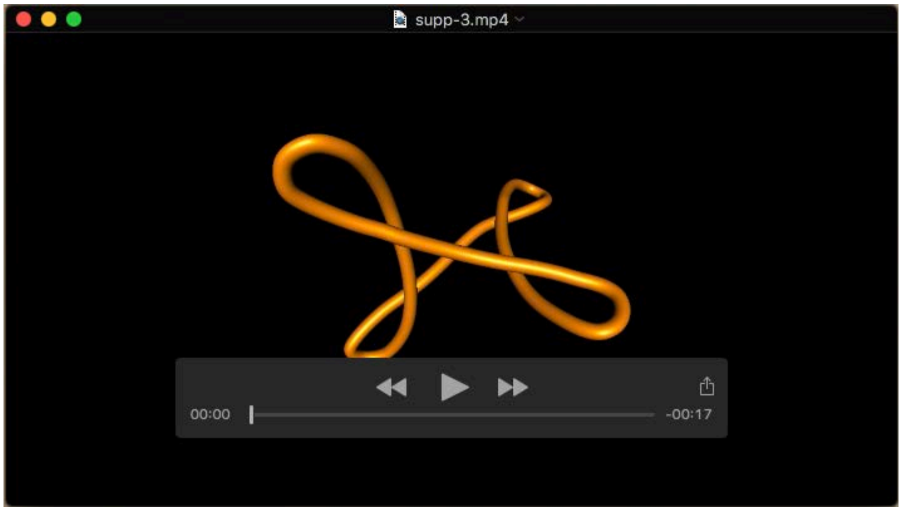

Movie 3: 4-string tangle model for DNA bound by condensin. The 4-string tangle model contains one postive supercoil in each of the four branches and one negative supercoil in the middle branch. As we rotate this model, the negative supercoil in the middle is no longer visible and we briefly see only three positive supercoils. As we continue rotating four positive supercoils appear. We again briefly loose one positive supercoil until we return back to the projection where we can view all four positive supercoils and the one negative supercoil in the middle branch.
